# Comparison of the effectiveness of antibody and cell-mediated immunity against inhaled and instilled influenza virus challenge

**DOI:** 10.1186/1743-422X-10-198

**Published:** 2013-06-19

**Authors:** Katie Rivers, Larry E Bowen, Jin Gao, Kevin Yang, John E Trombley, J Kyle Bohannon, Maryna C Eichelberger

**Affiliations:** 1 Division of Viral Products, OVRR, CBER, FDA, 8800 Rockville Pike, Building 29A 1D24, Bethesda, MD 20892, USA; 2 Southern Research Institute, Birmingham, AL 35205, USA; 3 Current address: Alion Science and Technology, NIEHS Inhalation Toxicology Facility, 5 Triangle Drive, P.O. Box 12313, Durham, NC 27709, USA

**Keywords:** Influenza, Aerosol, Inhalation, Instillation, Mouse, Antibody, CD8+ T cell, Immunity

## Abstract

**Background:**

To evaluate immunity against influenza, mouse challenge studies are typically performed by intranasal instillation of a virus suspension to anesthetized animals. This results in an unnatural environment in the lower respiratory tract during infection, and therefore there is some concern that immune mechanisms identified in this model may not reflect those that protect against infectious virus particles delivered directly to the lower respiratory tract as an aerosol.

**Method:**

To evaluate differences in protection against instilled and inhaled virus, mice were immunized with influenza antigens known to induce antibody or cell-mediated responses and then challenged with 100 LD_50_ A/PR/8/34 (PR8) in the form of aerosol (inhaled) or liquid suspension (instilled).

**Results:**

Mice immunized with recombinant adenovirus (Ad) expressing hemagglutinin were protected against weight loss and death in both challenge models, however immunization with Ad expressing nucleoprotein of influenza A (NP_A_) or M2 resulted in greater protection against inhaled aerosolized virus than virus instilled in liquid suspension. Ad-M2, but not Ad-NP_A_-immunized mice were protected against a lower instillation challenge dose.

**Conclusions:**

These results demonstrate differences in protection that are dependent on challenge method, and suggest that cell-mediated immunity may be more accurately demonstrated in mouse inhalation studies. Furthermore, the data suggest immune mechanisms generally characterized as incomplete or weak in mouse models using liquid intranasal challenge may offer greater immunity against influenza infection than previously thought.

## Background

Annual influenza epidemics result in approximately 40,000 deaths in the USA and at least one million deaths worldwide [1,2]. Vaccination provides protection against illness when the antigens in inactivated split vaccines are antigenically-matched to the circulating influenza viruses, reflecting the activity of antibodies that neutralize virus infectivity and limit virus spread. Both hemagglutinin (HA) and neuraminidase (NA)-inhibiting antibodies are independent correlates of immunity [3]. However, cell mediated immunity contributes to virus clearance, with the number of IFN-γ secreting T cells correlating with the efficacy of live, attenuated influenza vaccine in children [4].

Efforts are currently being made to develop universal influenza vaccines that offer broad protection by using target antigens that are conserved across influenza A subtypes: these antigens include nucleoprotein (NP), Matrix (M), the conserved stem region of HA, neuraminidase (NA), and M2. Several publications show that adenovirus (Ad) provides a suitable vector for delivery of M2 and NP antigens [5-8]. Adenovirus expressing M2 is known to induce high M2-specific antibody titers that bind to a highly conserved region of M2 that is extracellular (M2e) as well as T cell responses in BALB/c mice. However, antibody responses are sufficient to protect against challenge with PR8 [9]. These antibodies do not act alone, relying on FcR + NK cells to kill the M2-expressing infected cells [10]. Immune responses to NP expressed by recombinant adenovirus include both antibody and CD8+ T cell responses, with the NP-specific CD8+ T cells and not antibodies contributing to virus clearance. While the lung parenchyma contains some NK [11] and memory T cells [12], these cell types are recruited in substantial numbers from the circulation. Recruitment is a response to chemokines produced by infected cells or activated macrophages – NK cells migrate toward a variety of soluble mediators expressed at infected or inflammatory sites (reviewed in [13]) and there is evidence that IL-15 is responsible for recruitment of influenza-specific CD8+ T cells to the infected lung [14].

The animal models used in preclinical studies of these vaccines include mice, guinea pigs and ferrets [15-17]. These animal models typically utilize an intranasal or intra-tracheal route of virus challenge, with virus delivered in a liquid suspension to anesthetized animals so that natural reflexes to swallow or sneeze are avoided. The volume administered is substantial, ensuring that virus is deposited in the lower respiratory tract. This large challenge volume is likely to impact normal lung physiology, infection kinetics, as well as the subsequent induction of innate responses and recall of B and T cell memory. This presents conditions throughout the respiratory tract that are not representative of natural infection and we hypothesize, may result in reduced capacity of some immune mechanisms to protect against virus challenge. Influenza can be transmitted by aerosol and by direct contact with secretions or fomites [18]. While it is debated which mechanism is predominant, infectious aerosols are likely a common means of transmitting influenza because very small droplets that are formed when individuals sneeze or cough, can be inhaled and deposited in the lower respiratory tract [19]. A review of recent animal and human studies point to the importance of aerosolization in influenza transmission [20].

Early mouse studies show that fewer infectious units are required to infect mice by inhalation than instillation [21] and that for direct deposition in the lower respiratory tract, the aerosolized droplets should be <10 μm in diameter [22]. It is reasonable to expect that greater pathology and more severe disease would be observed when virus is administered as an aerosol compared to an instilled liquid suspension. This is indeed the case [23] with inoculation of a virus aerosol resulting in replication in Type II pneumocytes, the cell type observed as targets of natural human influenza infection, supporting the concept that inhalation of virus particles is likely an important mode of transmission in humans [24].

The aggravated disease experienced when virus is administered as an aerosol is clearly evident for A/Vietnam/1203/2004 (H5N1) which is not lethal by instillation in ferrets, but when administered as an aerosol is neurotropic and more pathogenic in this species [25]. Differences in the viral load, infectivity and pathogenesis when animals are challenged by different methods raise concern that a different magnitude or quality of the immune response would be required to control infection initiated by inhaled or instilled virus. We have established a mouse influenza inhalation model using nose-only exposure to virus contained within aerosol particles < 2 μm mass median aerodynamic diameter, and determined the LD_50_ of mouse-adapted A/PR/8/34 (PR8) delivered under these conditions [26]. We demonstrated that approximately 10-20 fold less aerosolized virus results in death than instilled virus, a difference previously noted by others [27]. In this study we compare the ability of different vaccination strategies to protect mice against a lethal dose of inhaled and instilled influenza virus.

## Results and discussion

### Immunization with live virus and recombinant adenovirus (Ad) expressing influenza proteins

BALB/c mice were exposed intranasally to a sublethal dose of PR8 (H1N1) or a heterologous reassortant H3N2 virus X-31, or vaccinated intramuscularly with recombinant adenovirus (Ad) expressing HA, M2, and NP of influenza A viruses (Ad-NP_A_), or NP of influenza B viruses (Ad-NP_B_). The latter vaccine group as well as naïve mice served as negative controls since these mice should not be protected against challenge with PR8. Three weeks after vaccination, the mice were bled and hemagglutination inhibition (HAI) antibody titers measured. Mice immunized with Ad-HA, but not Ad-M2 or Ad-NP had HAI titers of approximately 1:100 showing that this vaccination strategy is effective at inducing an antibody response. The capacity of Ad-M2 and Ad-NP to induce M2-specific antibodies and NP-specific CD8+ T cells, has been established by others using these same recombinant adenovirus preparations [5,6,28]. HAI antibodies reactive with PR8 were also present in the sera of mice exposed to sublethal dose of PR8 (HAI titers of approximately 1:600), but not following X-31 (H3N2) exposure. As others have reported, NP-specific CD8+ T cells are activated after live influenza A virus infection, and are recruited in substantial numbers to the lungs following challenge with virus that is of a different subtype [29].

### Virus replication in the lungs of vaccinated mice is similar 4 days after inhaled and instilled PR8 challenge

Five weeks after virus exposure or vaccination, equal numbers of mice (n = 10) in each vaccine group were exposed to PR8 as an inhaled aerosol or as an instilled liquid suspension. Aerosol challenge was conducted as described in Methods and Materials, using a nose-only exposure system without sedating the mice. In contrast, instillation was conducted by placing drops of a virus suspension onto the nares of anesthetized mice. The same lethal dose (100 LD_50_) was used; since BALB/c mice die when 10-fold lower amount of PR8 is inhaled compared to intranasal instillation [26], the amount of virus used in the inhalation challenge was approximately 10-fold less than in the instillation challenge.

**Figure 1:**
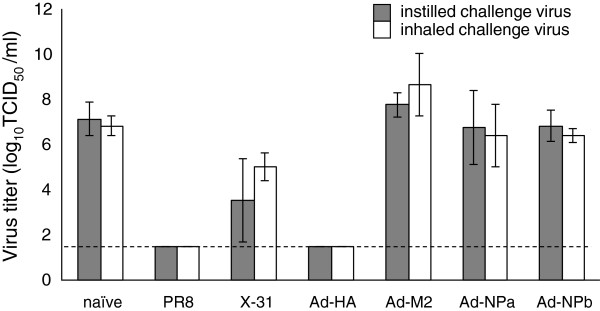
**Comparison of virus titers in the lungs of vaccinated mice after instillation and inhalation PR8 challenge.** Geometric mean virus titers (TCID_50_/ml) are shown for mice challenged by instillation (grey bars) and inhalation (white bars). Virus titers were determined for lungs (5 mice per group) collected on day 4 post-challenge, and homogenized in 1 ml of serum-free medium. Error bars indicate standard deviation and the dashed line is the limit of quantification for this assay.

Five mice in each group were sacrificed 4 days after challenge and lungs removed for virus titration; the clinical signs of disease and mortality were monitored in the remaining animals. These results show similar amounts of virus in the lungs of challenged mice, regardless of the challenge method (Figure 1). At this time point, virus was not detected in the lungs of mice previously exposed to PR8 or vaccinated with rAd-HA. There was a significant reduction in lung virus titers for mice previously exposed to the heterologous X-31 virus, providing evidence that heterosubtypic immunity induced by previous live virus infection, provides similar protection against inhaled and instilled virus. However, immunization with Ad-M2 and Ad-NP_A_ did not reduce day 4 virus titers after either inhalation or instillation.

### Complete immunity against both inhaled and instilled influenza in mice previously exposed to live homologous or heterologous virus, or vaccinated with Ad-HA

**Figure 2:**
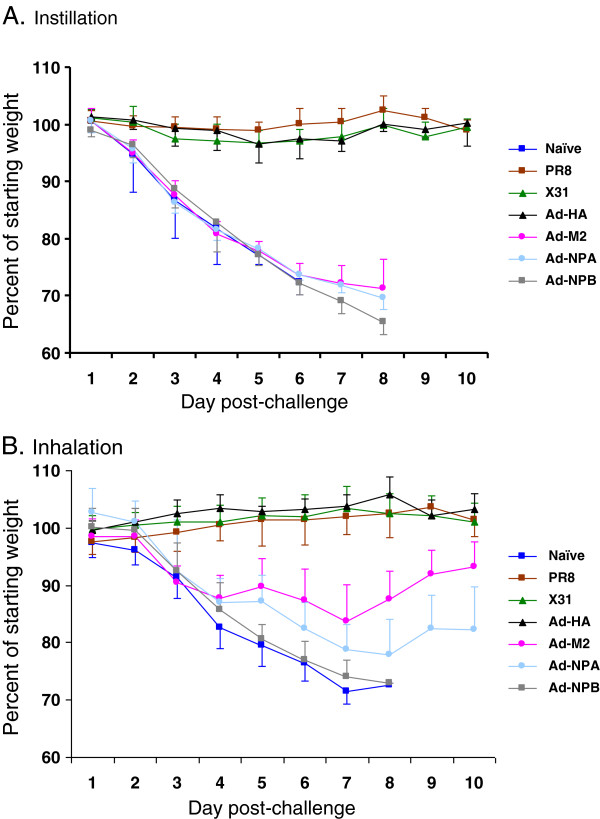
**Mice immunized with rAd-M2 and rAd-NP are protected against weight loss when virus is inhaled, but not when virus is instilled.** The average percent of starting weight for each group is shown for mice that were challenged by (**A**) instillation and (**B**) inhalation. Mice in each group (n = 5) were weighed individually on each day after challenge. Each graph shows the percent group mean change in weight relative to the baseline body weights, with error bars indicating the standard deviation for the group.

**Figure 3:**
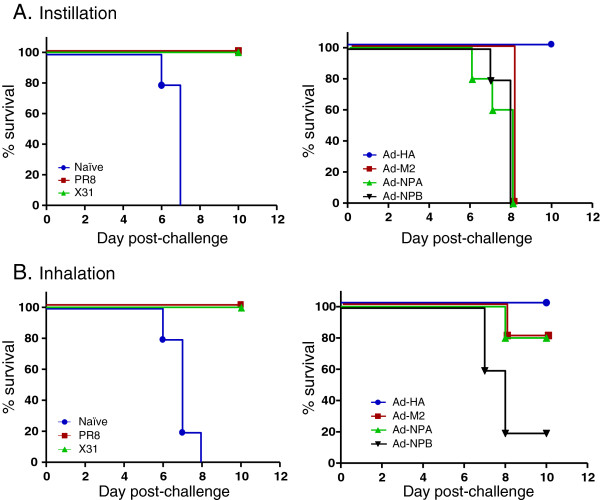
**Mice immunized with rAd-M2 and rAd-NPA survive inhaled, but not instilled, PR8 challenge.** Survival of immunized mice is shown for (**A**) instilled, and (**B**) inhaled virus challenge. Mice in all groups (n = 10) were challenged on the same day; to more easily visualize differences, naïve, PR8 and X-31-exposed mice are shown in graphs on the left hand side, and groups vaccinated with adenovirus recombinants expressing HA, M2, NP_A_ and NP_B_ are shown graphically on the right hand side. Since 5 mice were sacrificed in each group on day 4, survival of the remaining 5 was monitored to the end of the study (12 days post challenge). A key indicating the color code for each group is provided on the figure. Survival against both instilled or inhaled virus challenge was significantly greater for mice previously infected with either PR8 or X31 than naïve mice (Mantel-Cox test, p < 0.001); similarly survival of mice immunized with Ad-HA was greater than Ad-NP_B_-immunized mice (control group) when challenged with instilled or inhaled virus challenge (p < 0.001). The difference in survival between Ad-NP_B_-immunized and Ad-M2 or Ad-NP_A_-immunized mice was not statistically different when challenged with instilled virus, but trended towards significance (p = 0.054) for mice challenged by inhalation.

Mice that had previously been exposed to infectious PR8 or that had been immunized with rAd-HA had no virus in their lungs 4 days after either inhalation or instillation of 100 LD_50_ of the homologous virus (Figure 1). Not only were these mice protected from infection, they were fully protected against weight loss (Figure 2) and death (Figure 3). This protection correlates with the presence of HA inhibiting antibodies that are known to neutralize virus infectivity.

Although some virus replication was measured in the lungs, mice previously exposed to heterologous live X-31 virus were completely protected against weight loss (Figure 2) and death (Figure 3) after both inhaled and instilled virus challenge. These mice did not have antibodies that inhibit either HA or NA activities of the challenge virus, PR8 (not shown). As reported in numerous studies of heterologous protection, this protection is usually mediated by influenza-specific CD4+ and CD8+ T cells that kill infected cells [5,29], although antibodies to conserved antigenic epitopes, such as M2e, may also contribute to heterosubtypic immunity [9,30,31].

### Immunity against inhaled but not instilled influenza in mice vaccinated with recombinant Ad-M2 or Ad-NP_A_

Ad-M2, Ad-NP_A_, and Ad-NP_B_-immunized mice lost weight at the same rate as the naïve mice after challenge with a liquid suspension of PR8 (Figure 2). These groups of mice all died by day 8 post-challenge (Figure 3). Interestingly, this was one day later than the challenged naïve mice died, suggesting some benefit of the Ad-M2, Ad-NP_A_ and Ad-NP_B_ immunizations. In contrast, weight loss after aerosol challenge of the Ad-M2 and Ad-NP_A_-immunized mice slowed 3 days after aerosol challenge, although their weights remained significantly less than the Ad-HA group (p < 0.001, two-way repeated measures ANOVA) and neither group regained all weight by day 10 post-challenge. The M2-immunized mice were partially protected from this aerosol challenge, with survival of 4 of 5 mice whereas all the naïve mice succumbed to infection by day 8 post-inhalation challenge. Survival between Ad-NP_B_-immunized and Ad-M2 or Ad-NP_A_-immunized mice was not different, but trended towards significance (p = 0.054). As noted for challenge with instilled virus, immunization with Ad-NP_B_ provided a small but consistent benefit, with 1 of the 5 Ad-NP_B_-immunized mice surviving aerosol challenge. Repeat experiments with larger numbers of mice per group are needed to verify the significance of these findings.

Since the mice used in this study were from the same birth cohort and immunized with the same vaccine preparations at the same location and time, differences in protection against instilled and inhaled virus could reflect differences in virus load and/or differences in the capacity of the immune response to protect these different challenge conditions. In mice, lung virus titers are usually not reflective of input dose and therefore we were not surprised by the similar virus loads measured 4 days after challenge with inhaled and instilled virus (Figure 1). However, since the LD_50_ of inhaled virus was 10-fold less than the instilled virus, the number of infectious particles (approximately 870 vs 5160 PFU/mouse [26]) was clearly different and therefore an additional experiment was conducted to test whether the initial dose explained the difference in protective capacity. Ad-M2 and Ad-NP_A_-immunized mice were therefore challenged by intranasal instillation with similar total infectious units of PR8 as had been inhaled. This amount of virus was therefore equivalent to 10 LD_50_ in the instillation model.

**Figure 4:**
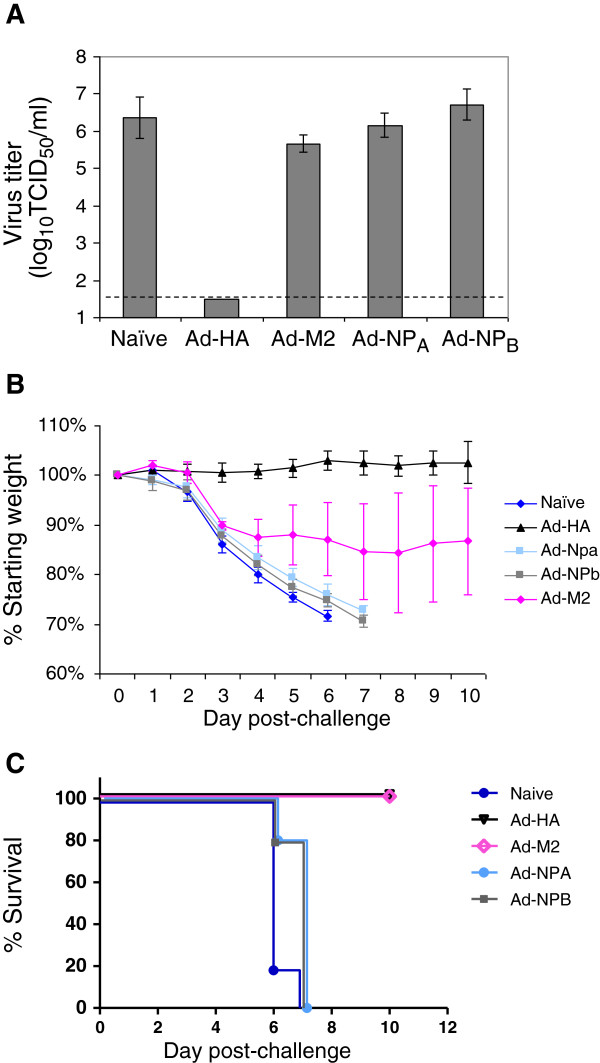
**(A) Virus titers, (B) weight loss and (C) mortality of 10LD**_**50 **_**instilled PR8.** Groups of mice (n = 10) were immunized as previously and challenged by instillation of PR8. Four days after challenge, 5 mice in each group were euthanized to allow titration of virus in the lungs, the weight and survival of the remaining mice was monitored until day 12. (**A**) shows geometric mean virus titers, together with the standard deviation; the dashed line is the limit of quantification for this assay. (**B**) shows the percent reduction in weight relative to the baseline body weights, with error bars showing standard deviation. A key for the groups is shown to the right of this graph. (**C**) shows percent survival of mice in each group. A key for these groups is shown to the right of the graph. Survival of mice vaccinated with Ad-HA and Ad-M2 was significantly greater (Mantel-Cox test, p = 0.0016) than mice immunized with Ad-NP_A_ or Ad-NP_B_. The latter 2 groups were not different from one another or naïve mice.

This experiment included a group challenged by instillation with 100 LD_50_ PR8, repeating the first experiment. Weight loss and survival of groups challenged with 100 LD_50_ PR8 were the same as noted previously. As for this high challenge dose, the mice immunized with Ad-HA were fully protected against virus replication, weight loss and death when challenged with the low virus dose (Figure 4). The rate at which mice in the Ad-NP_A_-immunized group lost weight was similar to that of naïve and Ad-NP_B_-immunized mice, and as observed when the Ad-NP_A_ mice were challenged with a higher dose of instilled PR8, all mice in this group died by day 8 post-challenge. In contrast, although there was variability in the weight loss for Ad-M2-immunized mice, the average percent weight loss was less after challenge with the low dose compared with the higher dose (Figure 4B vs Figure 2A), and all mice survived (Figure 4C vs Figure 3A). This result demonstrates that M2-mediated immune protection against instilled virus is dependent on the dose of virus inoculated. Clearly the effectiveness of M2 and NP-specific immunity, but not HA-specific immunity, is compromised when mice are challenged by instillation. We therefore predict that instillation impacts the environment in the lower respiratory tract, influencing the function of some immune mechanisms. This appears to correlate with the type of immunity that contributes to protection: HA-specific antibodies induced by Ad-HA protected against both inhalation and instillation challenge, while M2-specific antibodies that rely on NK cells to kill infected cells provided protection against infection that was dependent on the challenge dose, and NP-specific CD8+ T cells, the effector cells induced by Ad-NP_A_-immunization, provided some protection against inhaled but not instilled virus challenge. This suggests that for the immunization regimen we have used, antibody-mediated immunity is effective regardless of challenge method, while cell-mediated immunity is more effective against virus that is inhaled than instilled into the lungs.

Challenge by inhalation of very small virus-containing aerosol particles has a lower LD_50_ dose [26] and therefore a simple explanation for the difference in protection was that a smaller number of virions enter the lower respiratory tract of mice inhaling virus than in mice exposed to instilled virus. We tested this idea by inoculating mice with 10-fold less instilled virus. Our results showed this was not the case - mice immunized with Ad-NP_A_ were not protected against challenge with the equivalent number of inhaled and instilled virus particles.

Our results are reflective of published reports showing that vaccination strategies eliciting influenza virus-specific CD8+ T cells do not protect mice against intranasal challenge with a virus suspension [32], even though activated CD8+ T cells are capable of protecting mice when transferred directly to the lung [33]. A number of reasons have been proposed for this discrepancy [32], one being that compared to transfer of influenza-specific effector T cells, few influenza-specific CD8+ T cells traffic to the lung. Our findings of protection against inhaled, but not instilled PR8 in Ad-NP_A_-vaccinated mice suggests that the milieu in the lower respiratory tract is dependent on the method of challenge, with recruitment and/or effector function of CD8+ T cells supported when aerosolized virus, but not a liquid suspension, is used to challenge the mice. We propose that this is potentially due to a difference in the chemokine gradient needed for recruitment of NK and CD8+ T cells. It is feasible that the liquid instilled into the lower respiratory tract during challenge prevents establishment of the optimal chemokine gradients by direct dilution of these soluble mediators, or alternatively, activation of physiologic responses such as beating of cilia or secretion of mucins remove chemokines or prevent effective contact between T cells and the infected epithelium. The hypothesis that instillation disrupts chemokine gradients could be tested by comparing the concentrations of these soluble factors in vivo, and measuring the kinetics or magnitude of NK or T cell recruitment to the infected lung.

While a difference in chemokine gradient provides a potential reason for cell-mediated protection against inhaled but not instilled virus, there are other explanations to consider. For example, differences in the cell-types infected or innate mechanisms activated may result in more effective clearance of inhaled than instilled virus by influenza-specific CD8+ T cells. Future experiments should therefore examine the histopathology of lungs from infected mice to determine the number and types of inflammatory cells recruited to the lungs, and to compare extent of infection, cell types infected, and cellular damage as viral or immune pathology could also contribute to the differences in protection against instilled and inhaled virus. Other factors that warrant testing include the potential impact of isoflurane on instilled virus infectivity and function of responding cells [34], and possible differences in immune responses induced in the upper respiratory tract by instilled and inhaled virus. Further experiments are clearly needed to identify the reasons for the difference in Ad-NP_A_-induced protection against instilled and inhaled virus.

Our results show that mice were fully protected against lethal PR8 challenge by prior exposure to the heterosubtypic virus, X31. While antibodies contribute to cross-protection [30], cell-mediated immunity plays a significant role in immunity with large numbers of memory NP-specific CD8+ T cells recruited to the lungs [35]. Our results showing ineffective protection of Ad-NP-immunized mice against instilled virus in our model using a single vaccine dose suggest that the NP-specific immune response, and possible also protection against instilled virus, could be improved by administration of additional doses or in combination with adjuvant.

In summary, we compared protection against inhaled and instilled virus in mice previously exposed to homologous and heterosubtypic virus, or previously immunized with recombinant adenoviruses expressing HA, M2 or NP. Our results show similar protection against inhaled and instilled challenge for mice that were previously infected or vaccinated with Ad-HA, but not for mice immunized with Ad-M2 or Ad-NP_A_. In these latter groups, there was significant protection against inhaled, but not instilled PR8. These results suggest immune mechanisms often characterized as weak, such as NK or CD8+ T cell-mediated killers, may play a more significant role in protecting against disease and death than previously suggested from studies in which mice were challenged by instillation of a liquid virus suspension. Our data suggest that animal models using aerosol challenge may be a suitable approach to evaluate the potential efficacy of influenza vaccines for which cell-mediated responses are the correlate of immunity.

## Methods and materials

### Virus and vaccine preparations

A/PR/8/34 (H1N1) and A/HK/68 × 31 (H3N2, X31) virus stocks were prepared by inoculating 9-11 day old embryonated chicken eggs. The allantoic fluid was harvested 60-72 hr post-inoculation, cell debris removed by centrifugation and aliquots stored at -80°C until further use. Infectious titer of each stock was determined by titration on MDCK cells in a standard TCID_50_ assay as previously described [36]. Mice were rendered immune to the challenge virus (positive control) by immunization with a sublethal dose of live virus, with intranasal delivery under isoflurane anesthesia. Mice were immunized with individual influenza proteins using recombinant adenoviruses (rAd) that were administered intramuscularly (50 μl containing 10^10^ virus particles). The Ad5-ΔE1ΔE3 vector was used to express HA (rAd-HA), M2 (rAd-M2) and NP (rAd-NP). rAd-M2 was kindly provided by Dr Suzanne Epstein (CBER, FDA), and rAd expressing nucleoprotein (NP) was kindly provided by Dr Gary Nabel (VRC, NIH). The rAd-NP_A_ construct induces NP-specific CD8+ T cells in BALB/c mice [5,9]. The HA gene of PR8 was amplified using primers Hind III-H1 and Bam HI-H1, and the Hind III/Bam HI product inserted into pVQpacAd5CMVK-NpA (ViraQuest, North Liberty, IA). The construct was used to generate rAd expressing HA, by transfecting the construct into human embryonic kidney 293 cells that stably express E1A and E1B genes. Bulk rAd stocks were produced by ViraQuest, Inc. The recombinant adenovirus vectors expressing influenza A NP and consensus M2 that we used in our study have been described previously [8,9]. All virus stocks were stored in aliquots at -80°C.

### Mice and study design

Female BALB/c mice were purchased from The Jackson Laboratories (Bar Harbor, ME) and housed at Center for Biologics Evaluation and Research (CBER) where they were immunized. Mice that were subjected to nose-only inhalation challenge were shipped to Southern Research Institute (SR, Birmingham, AL) approximately 1 week before the start of the study. Cage size and animal care conformed to the guidelines prescribed in the Guide for the Care and Use of Laboratory Animals, the U.S. Department of Agriculture through the Animal Welfare Act.

Mice were challenged by instillation at CBER, and nose-only inhalation at SR. All experiments were performed under protocols 2006-22 and 10-217 approved by the Institutional Animal Care and Use Committees at CBER and SR, respectively. The mice were approximately 15 weeks of age and weighed between 19 and 26 grams at the time of virus challenge by inhalation or instillation.

### Inhalation challenge system

The murine inhalation challenge system consisted of six components: a compressed air source, a bioaerosol delivery line, a 24-port radial nose-only inhalation challenge plenum, a bioaerosol characterization platform, an air handling station, and an exhaust platform. The bioaerosol delivery line consisted of a Collison 3-Jet Nebulizer (BGI Inc., Waltham, MA), a radial in-line aerosol mixer (In-Tox Products, LLC; Albuquerque, NM), and a filtered air passive dilutor. The radial nose-only inhalation challenge plenum (In-Tox Products) was fitted with Positive Flow-By™ restraint tubes (In-Tox Products) and isoaxial sample collection ports that interfaced with the bioaerosol characterization platform. The bioaerosol characterization platform included air sampling impingers, Model 7541 (Ace Glass, Inc., Vineland, NJ), and an Aerosol Particle Sizer™ Spectrometer (APS, TSI Inc., Shoreview, MN). The air handling station interfaced with the bioaerosol delivery line, the bioaerosol characterization platform, and the exhaust platform and consisted of computer regulated gas flow and pressure controllers (Alicat Scientific, Inc., Tucson, AZ). The exhaust platform consisted of HEPA filters, a differential pressure magnehelic, and a vacuum pump. The bioaerosol delivery line, inhalation challenge plenum, and bioaerosol characterization platform were placed inside a SterilGARD^®^ III Advance™ (Baker Company; Sanford, ME) biological safety cabinet (BSC). The inhalation challenge plenum was maintained at a slightly negative pressure relative to the BSC. The BSC was maintained at a slightly negative pressure with respect to the Animal Biosafety Level-2 laboratory. Temperature and relative humidity within the inhalation challenge plenum were monitored using a Humidity Temperature Meter (Omega Engineering, Stamford, CT). Inhalation challenge plenum oxygen levels were monitored continuously during all challenges with a Model 5800 Intelligent Oxygen Monitor (Hudson RCI, Durham, NC).

Prior to conducting inhalation challenges, the system was characterized using PR8 virus [26]. Characterization included achieving and maintaining a target range of aerosol concentrations as determined by plaque assay analysis of impinger samples, and the demonstration of the aerosol particle size distribution of the challenge aerosol determined by APS analysis.

### Virus challenge

Inhalation of virus: On three consecutive days prior to inhalation challenge, mice were trained in the nose-only inhalation restraint tubes. On the day of challenge, a Collison 3-Jet Nebulizer was filled with virus stock suspension and connected to the bioaerosol delivery line. The dilution of virus needed to deliver 100 LD_50_ had previously been determined [26]. A pre-spray nebulizer suspension sample was collected to confirm that the correct virus dilution had been prepared. Mice were placed in nose-only restraint tubes and connected to the inhalation challenge plenum using Positive Flow-By™ nose cones (In-Tox Products, LLC; Albuquerque, NM). Groups of mice were exposed to nebulized virus for 30 minutes. The start of the challenge period (T = 0) began once the nebulizer was activated and set at 30 psi. Inhaled dose (approximately 870 PFU) was calculated as the product of aerosol concentration, murine minute ventilation (0.062 L/min [37]), and challenge duration.

Instillation of virus: Groups of mice were anesthetized by exposure to 3% isoflurane in the presence of 3% O_2_ and then inoculated with 30 μl of PR8 diluted in PBS (approximately 5,160 PFU), by applying droplets of the suspension to both nares.

All mice were observed twice daily during quarantine and study periods for signs of morbidity and mortality. Body weights were recorded daily. Animals that had lost ≥25% of their starting weight were euthanized. These mice were moribund, with hunched posture, inactivity and no response to handling. Survival curves were plotted taking into account both euthanized and animals found dead.

### Statistical analyses

Statistical analyses for body weight data were performed using the Provantis automated data collection system (Instem; Staffordshire, UK). Differences in weight between groups were determined by two-way repeated measures ANOVA (Microsoft Excel 2003, Redmond, WA) and differences in survival were determined by log-rank Mantel-Cox test using GraphPad PRISM 5 software. The mean, standard deviation, and coefficient of variance were calculated for aerobiology data using Excel or SigmaPlot (Systat Software, Inc., San Jose, CA) when appropriate.

### Virus titration

The 50% tissue culture infectious dose (TCID_50_) was determined by titration on MDCK cells (ATCC line CCL-34) following a standard procedure that was previously described [36]. Briefly, serial ten-fold dilutions of each lung homogenate were inoculated into quadruplicate wells of a 96-well plate containing a monolayer of MDCK cells. After 1 h in a CO_2_ incubator at 37°C, an equal volume of serum-free medium containing 3% BSA and TPCK-treated trypsin (5 μg/ml) was added. After 3 days incubation, the remaining cells were stained with crystal violet in gluteraldehyde. The TCID_50_ titer was defined as the inverse of the dilution that showed cytopathic effect in 50% of the wells.

## Abbreviations

TCID50: 50% tissue culture infectious dose; HA: Hemagglutinin; NA: Neuraminidase; M: Matrix; NP: Nucleoprotein; Ad: Adenovirus; LD50: 50% lethal dose; CBER: Center for Biologics Evaluation and Research.

## Competing interests

The authors declare that they have no competing interests.

## Authors’ contributions

MCE designed and supervised experiments, KR prepared virus stocks, vaccinated mice, and performed instillation challenge experiments; LEB developed aerosol challenge method and supervised inhalation experiments; KY assisted with mouse experiments and performed virus titrations; JG prepared recombinant plasmids for production of rAd and determined HAI titers; JET and JKB performed inhalation experiments; MCE wrote the manuscript. All authors read and approved the final manuscript.
